# Jaundice in Children

**Published:** 1901-03-30

**Authors:** 


### Hospital Clinics and Medical Progress.

### JAUNDICE IN CHILDREN.

In a lecture delivered at the Great Ormond Street Hospital for Sick Children Dr. George Still[1] took up the subject of jaundice in children, dealing more especially with the question of prognosis. Icterus neonatorum occurs in a large proportion, variously estimated at from 30 to 80 per cent. of all infants. This form of jaundice appears almost always within the first week. The urine and fæces remain normal in the milder cases. The child's health does not suffer, and after four or five days, or at most one or two weeks, the jaundice disappears. Now and again, however, jaundice coming on like icterus neonatorum persists for a much longer time, and yet in the end passes away; and these are anxious cases, for when an infant shows jaundice within a few days after birth and the jaundice persists more than three weeks, one's tendency is to think of syphilitic hepatitis, or of congenital obliteration of the bile ducts, in either of which conditions the prognosis would be extremely bad. One must, then, examine such cases very carefully before regarding them as exceptional cases of prolonged icterus neonatorum. As to the cause of icterus neonatorum, Dr. Still holds that it is obstructive, arising probably from increased viscidity of the bile, which in some cases has been demonstrated post-mortem.

In congenital obliteration of the bile ducts the prognosis is always bad. It is by no means always easy to distinguish these cases from those due to syphilis, for in both the jaundice may be present from birth; it becomes very intense and is associated frequently with hæmorrhages; the liver and spleen are enlarged and too firm; and the infant wastes and dies. It is to be noted that with this malformation jaundice is by no means always present at birth as one would expect to be the case.

In children beyond the age of infancy jaundice is commonly catarrhal in origin, such catarrhal jaundice being most common between the ages of two and six years. Almost always the child has been ailing for some days before the jaundice appears. Often it is cross and fretful, the appetite is bad, and almost invariably there is something abnormal in the state of the bowels—diarrhœa, constipation, or offensive stools. Vomiting commonly precedes or accompanies the onset of the jaundice—the urine becomes dark and the fæces pale. Often there is pain in the epigastrium and right hypochondrium. The liver also may be very tender and may be felt to be enlarged, extending an inch or more below the edge of the ribs. Slowness of pulse and itching, which are mentioned as occurring in catarrhal jaundice in adults, do not commonly occur in children. As a rule the jaundice passes off in ten days or a fortnight, but it may last longer. In some children repeated attacks of jaundice may occur, which are probably of a catarrhal nature, although they may strongly suggest calculi.

Among the more serious causes of jaundice in children beyond the age of infancy, one of the most often overlooked, because unexpected, is cirrhosis—not the intercellular cirrhosis of syphilis, but the form which in the adult is almost always attributed to alcohol, in which the surface of the liver is irregular with large "hob-nail" projections, and on section shows bright yellow rounded areas embedded in a stroma of grey fibrous tissue. The jaundice in such cases is generally very slight; indeed there may be no jaundice at all. The striking feature is the great enlargement of the spleen. Given a child with slight jaundice, a very large spleen, and a tendency to epistaxis or other hæmorrhages, and one has sufficient grounds for suspecting cirrhosis. If in addition the liver can be felt—and it may not be easily felt in these cases—its hardness and irregularity of surface may clinch the diagnosis. The children suffering from this condition are generally between the ages of four and fourteen years, and, in most cases, there is a fatal result within two or three years from the first appearance of the symptoms. It seems likely that in many if not in most of such cases there is no connection whatever with alcohol. Jaundice in children may also occur as a complication of heart disease, pneumonia, and tuberculosis—in the last two of which it is of very grave significance. One of its commonest associations is with heart disease, and although one's tendency is to consider such a complication as indicating "the beginning of the end," this is certainly not always so.

[1] Clinical Journal, Mar. 16.

